# Extended haematological follow-up after parenteral artesunate in African children with severe malaria

**DOI:** 10.1186/1475-2875-11-S1-P83

**Published:** 2012-10-15

**Authors:** Thierry Rolling, Dorothee Spahlinger, Saadou Issifou, Tsiri Agbeniyega, Peter G Kremsner, Gerd D Burchard, Benjamin Mordmueller, Jakob P Cramer

**Affiliations:** 1 Department of Internal Medicine, Section of Tropical Medicine, University Medical Centre Hamburg-Eppendorf, Hamburg, Germany; 2 Department of Clinical Research, Bernhard-Nocht-lnstitute for Tropical Medicine, Hamburg, Germany; 3 Medical Research Unit, Albert Schweitzer Hospital, Lambaréné, Gabon; 4 Kwame Nkrumah University of Science and Technology School of Medicine, Kumasi, Ghana; 5 Institute of Tropical Medicine, University of Tübingen, Germany

## Background

Mortality of severe malaria is significantly lower in patients treated with parenteral artesunate when compared to quinine. No significant side effects have been described from previous studies but follow-up periods have been limited.

First reports from Europe have shown cases of post-treatment haemolysis occurring two weeks after the first dose of parenteral artesunate. Furthermore, haemolysis was accompanied by a delay in adequate reticulocytopoiesis for up to two weeks. All patients developing post-treatment haemolysis had hyperparasitaemia. The aim of this study was to gain information about the incidence, clinical impact and pathophysiological background of this effect in the hyperendemic setting of sub-Saharan Africa.

## Materials and methods

This study was conducted as a substudy to the ongoing *Severe Malaria in African Children Follow-up study* and was implemented in Lambaréné, Gabon and Kumasi, Ghana. 50 patients were recruited per study site.

Patients were randomized to different treatment regimes of parenteral artesunate (3 doses of 4 mg/kg iv, im or 5 doses of 2.4 mg/kg im respectively). Haematological parameters and serum markers of haemolysis and erythropoiesis (lactate dehydrogenase, haptoglobin, bilirubine, soluble transferrin receptor and erythropoietin) were determined on days 0, 7, 14 and 28. The clinical impact of changes in haemoglobin was assessed.

## Results and conclusions

The preliminary results show a delay in the reticulopoietic response after treatment of severe malaria, which is accompanied by a slower recovery of haemoglobin levels in hyperparasitaemic patients (>100,000 parasites/µl), when compared to low parasitaemic patients. Hyperparasitaemic patients had a mean rise of 1.1 g/dL in Hb between days 0 and 28, while patients with low parasitaemia had a mean rise of 2.1 g/dL between days 0 and 28. First results on the potential pathophysiologic background including haemolysis and *I* or impaired erythropoiesis will be presented. In conclusion, there seems to an association between parasite levels and recovery of haemoglobin levels. Whether or not this effect is associated with malaria disease, treatment with parenteral artesunate or both is subject of further investigations.

